# The Hepatoprotective Effect of Sodium Nitrite on Cold Ischemia-Reperfusion Injury

**DOI:** 10.1155/2012/635179

**Published:** 2012-02-28

**Authors:** Wei Li, Zihui Meng, Yuliang Liu, Rakesh P. Patel, John D. Lang

**Affiliations:** ^1^Division of Transplantation, Department of Surgery, University of Washington Medical Center, Seattle, WA 98195, USA; ^2^Department of Hepato-Pancreatic Surgery, The Third Hospital of Bethune Medical College (China-Japan Union Hospital), Jilin University, Changchun, Jilin 130033, China; ^3^Department of Surgery, The University of Alabama at Birmingham, Birmingham, AL 35294, USA; ^4^Department of Pathology and Center for Free Radical Biology, The University of Alabama at Birmingham, Birmingham, AL 35294, USA; ^5^Department of Anesthesiology & Pain Medicine, University of Washington Medical Center, P.O. Box 356540, Seattle, WA 98195, USA

## Abstract

Liver ischemia-reperfusion injury is a major cause of primary graft non-function or initial function failure post-transplantation. In this study, we examined the effects of sodium nitrite supplementation on liver IRI in either Lactated Ringer's (LR) solution or University of Wisconsin (UW) solution. The syngeneic recipients of liver grafts were also treated with or without nitrite by intra-peritoneal injection. Liver AST and LDH release were significantly reduced in both nitrite-supplemented LR and UW preservation solutions compared to their controls. The protective effect of nitrite was more efficacious with longer cold preservation times. Liver histological examination demonstrated better preserved morphology and architecture with nitrite treatment. Hepatocellular apoptosis was significantly reduced in the nitrite-treated livers compared their controls. Moreover, liver grafts with extended cold preservation time of 12 to 24 hours demonstrated improved liver tissue histology and function post-reperfusion with either the nitrite-supplemented preservation solution or in nitrite-treated recipients. Interestingly, combined treatment of both the liver graft and recipient did not confer protection. Thus, nitrite treatment affords significant protection from cold ischemic and reperfusion injury to donor livers and improves liver graft acute function post-transplantation. The results from this study further support the potential for nitrite therapy to mitigate ischemia-reperfusion injury in solid organ transplantation.

## 1. Introduction

 Liver ischemia-reperfusion injury (IRI) is a major cause of primary graft nonfunction or initial function failure posttransplantation, both of which can ultimately lead to acute and/or chronic rejection. Moreover, the occurrence of significant IRI in marginal liver donors serves to limit the number of organs available for transplantation. Therefore, insights into therapies targeted toward attenuating liver IRI should assist in thwarting liver graft primary nonfunction or poor function, reduce the episodes of acute and chronic graft rejection, extend the usage of marginal donors, and thus aid in reducing the donor organ shortage. Interestingly, to date, clinically translatable mechanisms of liver IR have not yet been well characterized, resulting in a paucity of available therapies for prevention and treatment of liver IRI.

Nitric oxide (NO) is a free radical produced from L-arginine and is a versatile signaling mediator involved in a multitude of critical cellular events [1]. NO is also an important effector molecule produced by macrophage and dendritic cells (DCs) that is involved in immune regulation and host innate and adaptive immunity [2, 3]. NO has been found to attenuate liver IRI during transplantation through various mechanisms including reducing hepatocellular apoptosis and inflammatory tissue injury [4]. More recently, inhaled NO administered to human liver transplant recipients prior to transplantation and continued throughout the entire operative procedure significantly accelerated restoration of liver function by decreasing hepatocyte apoptosis [5]. While the underlying mechanisms of inhaled NO on liver IRI are largely unknown, the possibility of the anion nitrite, an oxidative product of NO metabolism that is increased in the circulation with NO breathing, playing a role has been suggested. Consistent with this concept, sodium nitrite has been shown to limit acute IRI in both murine heart and liver of warm IR and is associated with decreased incidence of myocardial infarction and hepatocyte apoptosis [6]. Underlying this protection are biochemical pathways that couple ischemia to nitrite reduction to NO, which then can mediate cytoprotective effects by multiple possible mechanisms [7–10]. Similarly, nitrite-dependent protection has been observed in other models of ischemic tissue injury encompassing all major organ systems [11]. In this study, we critically examined the protective effects of nitrite administration on liver IRI in a cold murine IRI model using different preservation solutions. Our study demonstrates that nitrite supplementation of either Lactated Ringer's (LR) or University of Wisconsin's (UW) solutions significantly reduced liver cold IR injury and protected hepatocytes from apoptosis posttransplantation.

## 2. Materials and Methods

### 2.1. Animals

Male C57BL/10 (B10; H2^b^) and C57BL/10 (C3H; H2^k^) mice, 10–12 weeks of age (The Jackson Laboratory, Bar Harbor, ME), were maintained in a specific pathogen-free facility of the University of Washington Medical Center. The mice were provided with Purina rodent chow and tap water* ad libitum. *Animal care was in compliance with our institutional animal care and use of committee-approved protocol, and with the “Guide for the Care and the Use of Laboratory Animals” published by the National Institutes of Health.

### 2.2. Reagents

Sodium nitrite was purchased from Sigma-Aldrich (cat no. S-2252, St. Louis, MO). UW solution was obtained from Life Center Northwest (Bellevue, WA, manufactured by Duramed Pharmaceuticals, Inc, Cincinnati, OH), and LR solution was from Baxter Healthcare Corporation (Deerfield, IL). ApopTag Peroxidase In Situ Apoptosis Detection Kit was from Millipore Corporation (Billerica, MA).

### 2.3. Nitrite Treatment Protocol

The livers were flushed via the portal vein with 5 mL of either cold LR or UW solution containing 20 units/mL heparin with or without sodium nitrite supplementation of 25–250 *μ*M and then were procured and stored in a sterile container containing 2 mL (equal mouse total body blood volume) perfusion solution for different time periods, from 1 hour to 48 hours, respectively. AST and LDH enzyme release from the preservants and the liver histology were examined at different time points. The time course curves were analyzed to determine the optimal concentration of nitrite. The livers preserved in the UW solution with or without nitrite supplementation were transplanted into syngeneic recipients after either 12 or 24 hours of cold preservation, respectively. The recipients of syngeneic liver grafts also received either sodium nitrite 250 *μ*mol in 2 mL total blood volume final concentration or equal amounts of phosphorus buffer solution (PBS) by i.p. injection just prior to liver transplantation. Liver tissue was obtained for histology and apoptosis assays at 10 minutes and 3 hours, respectively.

### 2.4. Liver Enzyme Assays

Samples from the liver preservants at 1, 6, and 12 hours in LR solution and 12, 24, and 48 hours in UW solution with the concentration range of nitrite from 0.25 *μ*M to 1000 *μ*m were assayed for ALT, AST, and LDH levels using a spectrophotometric method, respectively. The LR and UW solutions without anion nitrite supplementation were used as controls.

### 2.5. Liver Transplantation

Orthotopic liver transplantation (OLTx) with revascularization accomplished with a combination of suture and cuff techniques was performed between syngeneic strain combinations as described [3, 12]. Donor livers were preserved in the UW solution at extended times of 12 and 24 hours, with or without nitrite supplementation at 25 or 125 *μ*M, respectively. Liver biopsy was performed at 10 minutes and 3 hours postreperfusion, respectively.

### 2.6. Histological Analysis

Formalin-stored tissue specimens were embedded in paraffin and cut into 4 mm sections and then were examined by routine hematoxylin and eosin staining. Three samples from each group were analyzed under 10 high-power fields. The histology scores of liver tissue sections were determined by 2 independent persons in a blind manner according to the following scoring criteria: 0, no hepatocellular damage; (1) mild injury characterized by cytoplasmic vacuolization and focal nuclear pyknosis; (2) moderate injury with dilated sinusoids, cytosolic vacuolization, and blurring of intercellular borders; (3) moderate to severe injury with coagulative necrosis, abundant sinusoidal dilation, red blood cell extravasation into hepatic chords, and hypereosinophilia and margination of neutrophils; (4) severe necrosis with loss of hepatic architecture, disintegration of hepatic cords, hemorrhage, and neutrophil infiltration. Criteria to specifically evaluate peripheral mononuclear cell infiltration ((0) zero; (1) minimal; (2) mild; (3) moderate; (4) severe) were also used (Lang 2007).

### 2.7. TUNEL Staining

Apoptotic cells in frozen sections (4 *μ*m) were identified using the ApopTag Peroxidase In Situ Apoptosis Detection Kit (Millipore Corporation, Bilerica, MA) and followed according to the manufacturer's instructions, as described by (Li, 2008). Cryosections (4 *μ*m) were mounted on precleaned slides, air-dried overnight at room temperature (RT), and then fixed for 10 min at RT in 10% neutral buffered formalin (pH 7.4), followed by two washes (5 min each) in PBS. Endogenous peroxidase activity was quenched in 2% H_2_O_2_ before exposure to TdT enzyme at 37°C for 60 min. After washing in stop wash buffer (37°C, 30 min), anti-digoxigenin-peroxidase was added (RT; 30 min). AEC or DAB (ScyTek Laboratories, Inc, Logan, UT) was used for color development, and the sections were counterstained with hematoxylin. The numbers of apoptotic cells in liver sections were enumerated under light microscopy by numbers of apoptotic cells per 40 high-power fields in five sections per tissue per mouse (three mice per group).

### 2.8. Caspase-3 Activity Assay

A caspase-3 ELISA kit (BD Pharmingen, San Diego, CA) was used for *in vitro* determination of caspase-3 enzymatic activity in liver homogenates derived from mice in different groups. Briefly, supernatants were obtained from homogenized liver grafts with mammalian lysis buffer (Qiagen, Inc., Valencia, CA) including benzonase nuclease and protease inhibitor. Flat-bottom 96-well microtiter plates were coated with 100 *μ*L/wells of diluted capture antibody (BD Pharmingen). Nonspecific binding sites were blocked with blocking buffer. Plates were rinsed, and standards and samples of diluted supernatant (100 *μ*L each) were added, followed by the addition of 100 *μ*L/well working detectors (diluted detector antibody and streptavidin HRP in blocking buffer). After washing, the plates were incubated for color development; the reaction was terminated with 50 *μ*L of stopping solution. Plates were read at 450 nm in an ELISA reader. To determine the active caspase-3 concentration of samples, computer data reduction was employed, utilizing log-log regression analysis in a series of diluted caspase-3 standards. 

### 2.9. Western Blot Analysis

The antiapoptosis Bcl-2 protein expression in the liver tissue was determined by western blot assay. Proteins (50 *μ*g/sample) extracted from the liver tissue in SDS-loading buffer (50 mM Tris, pH 7.6, 10% glycerol, 1% SDS) were subjected to 12% SDS-polyacrylamide gel electrophoresis and transferred to PVDF membrane (Bio-Rad Laboratories, Hercules, CA). The gel was then stained with Coomassie Blue to document protein loading. The membrane was blocked with 5% dry milk and 0.1% Tween 20 (Bio-Rad) in PBS. The membrane was subsequently incubated with the primary antibodies at 4°C overnight. The primary antibody was a mouse monoclonal antihuman Bcl-2 antibody (Santa Cruz Biotechnology, Inc., Santa Cruz, CA). The membranes were developed according to the Amersham-enhanced chemiluminescence protocol. The beta-actin was measured as a loading control.

### 2.10. Statistical Analysis

The comparisons were made using Student's* t-*test. Probability (*P*) values ≤0.05 were considered statistically significant. Data is expressed as mean ± SE.

## 3. Results

### 3.1. The Protective Effects of Nitrite to Liver IRI Were Dose Dependent

**Figure 1:**
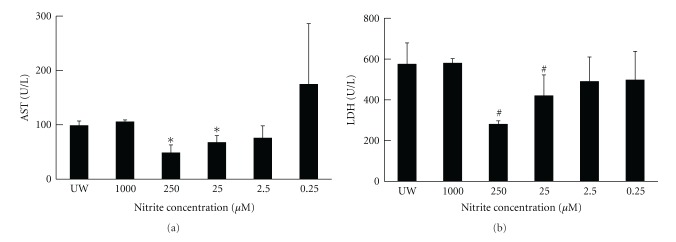
Nitrite-supplemented UW solution decreased hepatic graft enzyme release. UW solution supplemented with anion nitrite decreased hepatic graft enzyme release during 24 hours of cold ischemia. Solutions of 250 (AST—68 ± 12; LDH—420 ± 102) and 25 (AST—38 ± 4; LDH—280 ± 17) *μ*M anion nitrite resulted in the greatest reduction. Data are expressed as mean ± SE. ^#^ *P* < 0.05 for corresponding measurements.

To determine if nitrite supplementation could confer protection in the liver from cold ischemia injury, nitrite (0.25 *μ*M–1000 *μ*M) was added to UW solution. The livers enzymes AST and LDH were then measured from the preservation solutions at 24 hours. In contrast to the UW only control group, AST and LDH release was significantly reduced in livers supplemented with nitrite with maximal protection observed with 25 *μ*M and 250 *μ*M nitrite (AST—38 ± 4; LDH—280 ± 17) (Figure 1).

### 3.2. Nitrite Supplementation of LR or UW Solution Reduced Liver Damage from Extended Cold Ischemia Time

**Figure 2:**
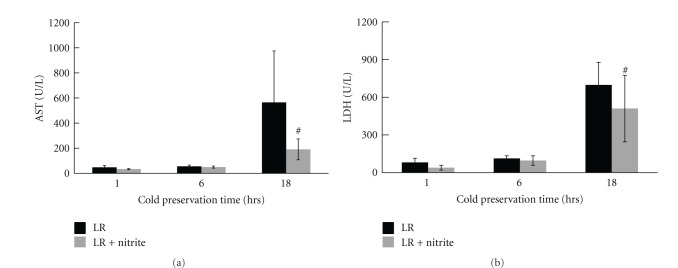
Nitrite-supplemented LR decreased graft enzyme release. LR supplemented with 25 *μ*M anion nitrite significantly decreased enzyme release at 18 hours (AST—599 ± 492 versus 178 ± 59; LDH—692 ± 269 versus 498 ± 272). Data are expressed as mean ± SE. ^#^ *P* < 0.05 for corresponding measurements.

**Figure 3:**
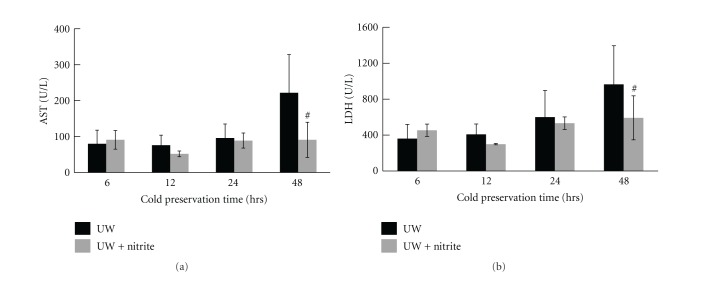
Nitrite-supplemented UW solution decreased graft enzyme release; UW solution supplemented with 25 *μ*M anion nitrite significantly decreased enzyme release at 48 hours (AST—221 ± .97 versus 76 ± 40; LDH—991 ± 420 versus 578 ± 292). Data are expressed as mean ± SE. ^#^ *P* < 0.05 for corresponding measurements.

We examined whether nitrite supplementation of either LR or a preservant (UW) could protect the liver from cold ischemia injury. B6 mouse livers were perfused with 5 mL cold Ringer's solution containing heparin 20 units/mL with or without nitrite (25 *μ*M) addition. Livers were then preserved in 5 mL of the above solution on ice for 1 hour, 6 hours, and 18 hours, respectively. AST and LDH levels were tested from the preservation solution from 3 livers pooled together in each group. AST was detected to have a slight increase after 1 and 6 hours of cold preservation and a significant increase after 18 hours of cold preservation in the LR control groups. The LDH level also increased significantly after 18 hours of cold preservation. Release of AST and LDH was significantly reduced in the nitrite-supplemented groups compared to LR only groups (Figure 2). Similarly, nitrite supplementation in the UW solution also reduced the release of liver AST and LDH significantly after 48 hours of cold preservation (Figure 3).

### 3.3. Nitrite Supplement in LR Solution Reduced Liver Damage from Extended Cold Ischemia Time

**Figure 4:**
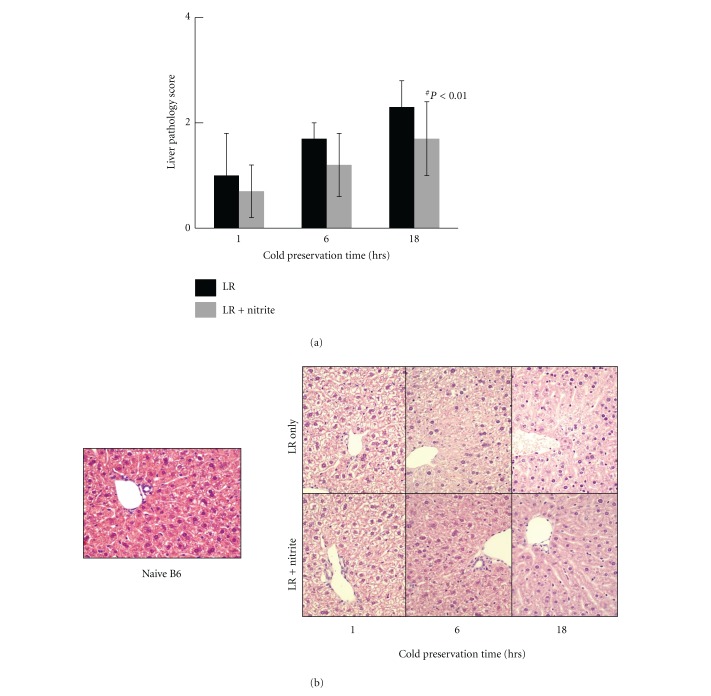
Liver pathology scores decreased with nitrite. Increased hepatocyte swelling, increased cytoplasmic vacuolization, nuclear pyknosis, sinusoidal dilatation, and focal necrosis in LR only group with maximal injury at 18 hrs are observed. These findings were significantly reduced at 18 hrs with 25 *μ*M of anion nitrite (2.4 ± 0.8 versus 1.7 ± 0.7). Data are expressed as mean ± SE. ^#^ *P* < 0.05 for corresponding measurements.

**Figure 5:**
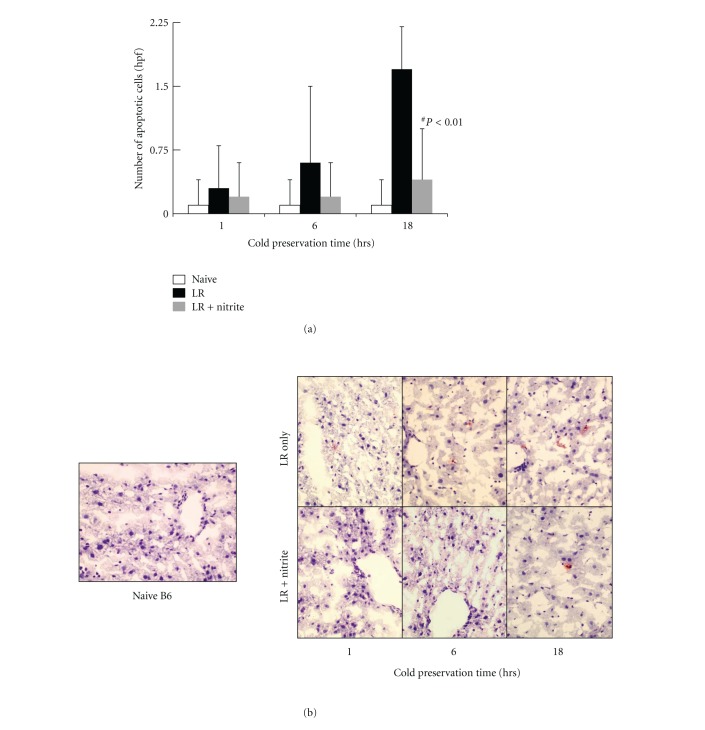
Hepatocellular apoptosis significantly decreased within LR-supplemented nitrite therapy. TUNEL evaluation revealed a significantly reduced number of hepatocellular apoptotic nuclei treated with nitrite at 18 hours (0.1 ± 0.3 versus 1.7 ± 0.4 versus 0.4 ± 0.5). Data are expressed as mean ± SE. ^#^ *P* < 0.05 for corresponding measurements.

**Figure 6:**
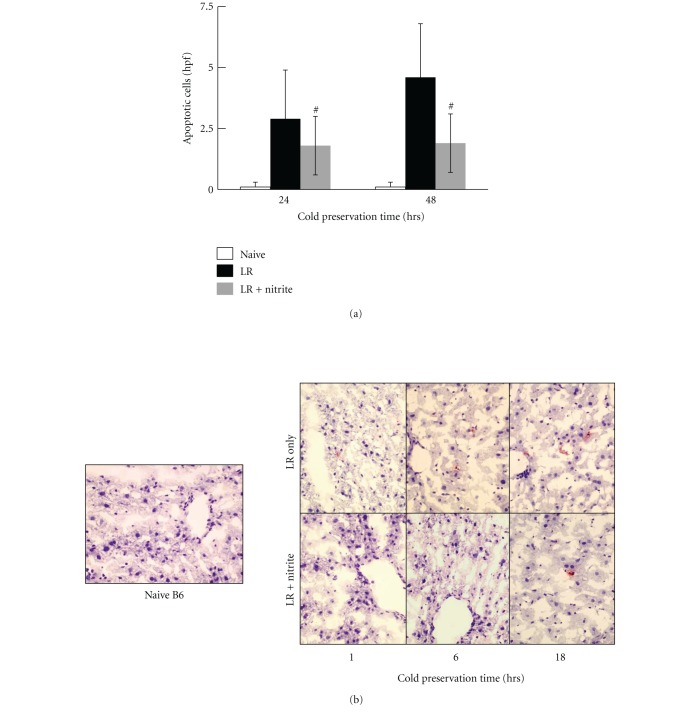
Nitrite reduced hepatocellular apoptosis during cold ischemia. Hepatocellular apoptosis was significantly reduced with 250 *μ*M of anion nitrite after 24 hours (0.2 ± 0.3 versus 2.9 ± 2.2 versus 1.7 ± 1.5) and 48 hours (0.2 ± 0.3 versus  4.3 ± 3  versus 1.8 ± 1.4) of cold ischemia. Data are expressed as mean ± SE. ^#^ *P* < 0.05 for corresponding measurements.

To examine the protective role of nitrite on hepatocytes during cold ischemia, liver samples were harvested from naïve B6 mice without perfusion, or from donor livers perfused and preserved with either LR solution only or LR plus anion nitrite supplementation at a concentration of 250 *μ*M after 1 hour, 6 hours, and 18 hours-cold preservation, respectively. Histological examination on H & E staining section revealed hepatocyte swelling, increased cytoplasmic vacuolization, nuclear pyknosis, sinusoidal dilatation, and focal necrosis in the LR only group after 6 hours of cold preservation, and becoming more severe at 18 hours. In contrast, addition of 25 *μ*M anion nitrite to the Ringer's preservation solution gave liver significant protection from cold ischemia injury during extended preservation period. The liver morphology remained essentially normal in those groups (Figure 4). Furthermore, livers preserved in the nitrite supplemented LR solution demonstrated a significant reduction of hepatocyte apoptosis after 18 hours of cold preservation (Figure 5). Similarly, nitrite supplementation of UW solution also reduced liver apoptosis after both 24 and 48 hours of cold preservation time (Figure 6).

### 3.4. Nitrite Modulates Caspase-3 and Bcl-2 Protein Expression

**Figure 7:**
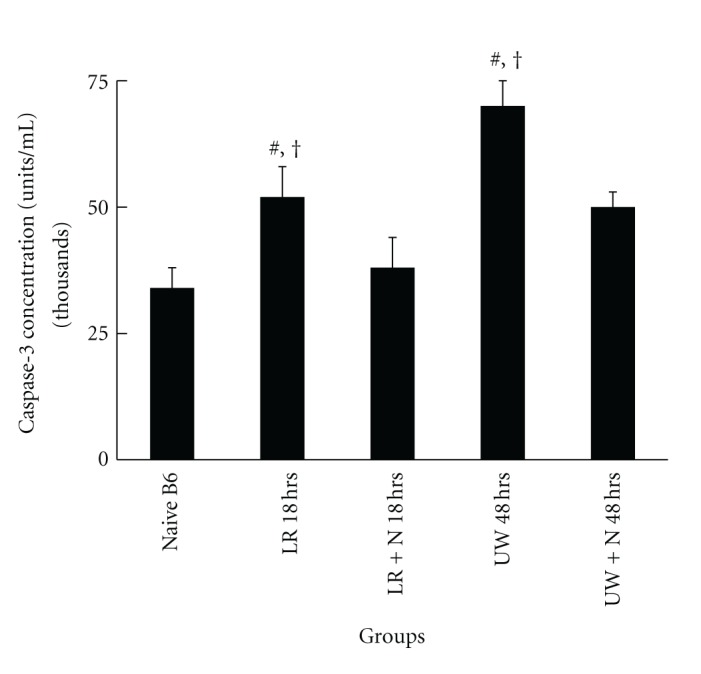
Caspase-3 concentrations decreased with nitrite supplemented solutions. Caspase-3 concentrations increased during cold preservation with both LR (18 hours) and UW solutions (48 hours) alone, but were significantly attenuated when the respective solutions were supplemented with 250 *μ*M of anion nitrite (LR—49.1 ± 3.7 versus LR + nitrite—38.6 ± 3.7; UW—71.4 ± 4  versus UW + nitrite 50.4 ± 3.1). Supplemented UW solution demonstrated the greatest attenuation. Data are expressed as mean ± SE. ^#^ *P* < 0.05 for corresponding measurements that compared groups LR and UW only to native B6 and ^†^ *P* < 0.05 for corresponding measurements that compared groups LR and UW only to LR and UW groups treated with anion nitrite.

**Figure 8:**
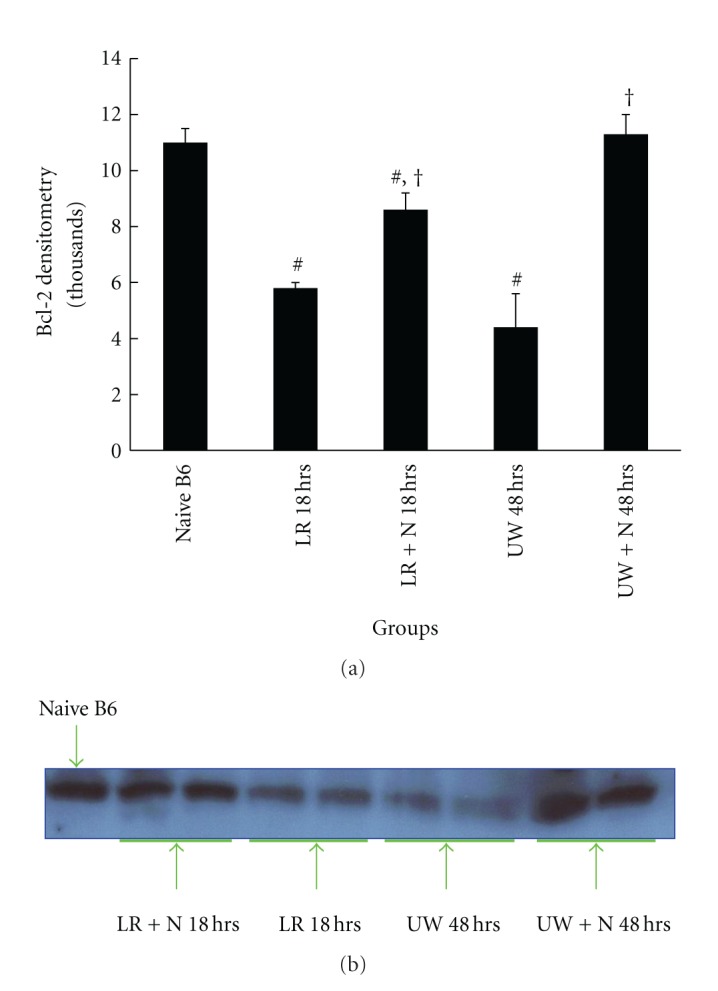
Nitrite-supplemented solutions mitigated reductions in Bcl-2 concentrations. Bcl-2 protein (pooled from 3 livers per condition) expression decreased during cold preservation with both LR (18 hours) and UW solutions (48 hours) alone but significantly increased when the respective solutions were supplemented with 250 *μ*M of anion nitrite (LR—5.9 ± 0.6 versus LR + nitrite—8.8 ± 1; UW—4.4 ± 1.3 versus UW + nitrite—10.8 ± 1.1). Supplemented UW solution demonstrated the greatest increase in protein expression. Data are expressed as mean ± SE. ^#^ *P* < 0.05 for corresponding measurements.

To determine the mechanisms by which nitrite decreases liver apoptosis under conditions of cold ischemia, caspase-3, a cysteine protease critical for executing apoptosis, and Bcl-2, a family of proteins demonstrated to reduce apoptosis were measured after varying cold ischemia times (18 and 48 hours). Caspase-3 in livers supplemented with 25 *μ*M nitrite in LR and UW solution during cold ischemia was significantly reduced at 18 (LR) and 48 hours (UW), respectively (Figure 7). The magnitude of caspase-3 reduction between the LR and UW preservation groups was similar (29.7 to 28.4%, resp.). Bcl-2 protein concentration determined by western blot analysis demonstrated a significant reduction in both LR and UW preservation solution alone. However, concentrations of both caspase-3 and Bcl-2 were restored to near or back to baseline values with anion nitrite supplementation (Figure 8).

### 3.5. Nitrite Reduced Liver Reperfusion Injury after Extended Cold Preservation Time in UW Solution

**Figure 9:**
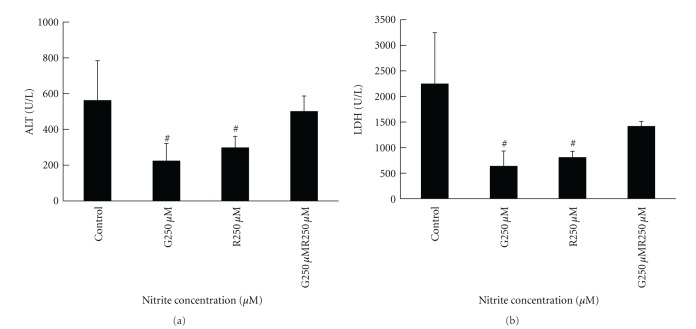
Nitrite administration to either the donor liver graft or recipient decreased hepatic enzyme release posttransplantation. Serum enzymes in only the nitrite-treated graft and recipient only groups decreased in syngeneic recipients 3 hours posttransplantation (ALT—580 ± 118 versus 210 ± 90 versus 229 ± 41 versus 493 ± 92; LDH-2228 ± 1019 versus 652 ± 380 versus 810 ± 182 versus 1375 ± 202). Data are expressed as mean ± SE. ^#^ *P* < 0.05 for corresponding measurements.

**Figure 10:**
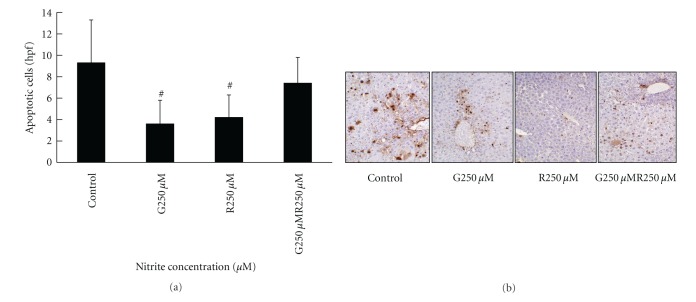
Nitrite reduced hepatocellular apoptosis in the donor liver graft and in the recipient after liver transplantation. Hepatocellular apoptosis was significantly reduced by anion nitrite in the donor liver graft and recipient only groups compared to control and combined treatment groups 3 hours posttransplantation (9.8 ± 4.1 versus 3.9 ± 2.4 versus 4.1 ± 2.6 versus 7 ± 3.9). Data are expressed as mean ± SE. ^#^ *P* < 0.05 for corresponding measurements.

To determine whether nitrite protected livers from IRI after liver transplantation, syngeneic liver transplantation from donor livers which were either preserved in UW solution plus 250 *μ*M nitrite for 12 hours or recipients that received 250 *μ*M i.p. nitrite treatments at the time of liver transplantation was performed. Liver enzyme release and apoptosis from the donated livers were examined 3 hours posttransplantation. Assessment of liver enzyme release revealed that nitrite supplementation in the preservation solution only and intraperitoneal treatment alone significantly decreased ALT and LDH release compared to the control group and the group receiving the combined treatment of both donor liver graft and recipient i.p. injection (Figure 9). Moreover, nitrite treatment of the donor liver only and recipient only significantly reduced hepatocyte apoptosis when compared to the control and combined treatment groups (Figure 10). 

## 4. Discussion

Previous studies in animal models of warm IRI utilizing nitrite as an anti-inflammatory therapy have demonstrated significant benefit. In this study, we chose to supplement solutions with varying concentrations of nitrite under conditions of cold ischemia and to assess potential anti-inflammatory effects of nitrite therapy on murine hepatic graft function. Our results show that nitrite-supplemented solutions during cold ischemia decreased hepatocellular injury and apoptosis during both cold ischemia and 3 hours posttransplantation.

Previous studies have shown that nitrite dose versus therapeutic benefit exhibits a U-shaped relationship [6]. A similar efficacy profile was observed with nitrite protection against cold-ischemia-mediated liver injury with 25–250 *μ*M affording maximal protection. Previous *in vivo* studies demonstrated protection against IRI injury in the liver with <10 *μ*M nitrite concentrations [6, 12] suggesting that liver grafts subjected to cold ischemia (compared to warm IRI) warrant higher concentrations of nitrite as 25 *μ*M consistently attenuated injury during cold ischemia alone, while 250 *μ*M anion nitrite was beneficial when administered to either the graft alone or recipient animal alone in animals 3 hours posttransplantation. Attenuation of cold ischemic injury by nitric oxide implicates the sinusoidal endothelial cells as a principal culprit as the injury orchestrator

Hepatic enzyme release significantly decreased when supplemented with nitrite demonstrating a more robust protective effective effect with UW compared to Ringer's solution as would be expected. The maximal protective effect peaked at 48 hours with the use of UW solution compared to 18 hours with Ringer's solution. Nitrite treatment to only the liver graft or recipient mouse was superior in reducing hepatic enzyme release compared to combination treatment of both graft and recipient. Interestingly, in this series of liver transplantation studies, the supplementation or administration of 250 *μ*M anion nitrite was required to exert a significant protective effect compared to 25 *μ*M used only during cold ischemia. The mechanisms underlying why the effective doses varied by 10-fold remain unclear.

Histological injury per standardized scoring system demonstrated reduced injury in liver grafts treated with nitrite. The injury was maximal at 18 hours, although not severe in either group. This observation is consistent with previous data corroborating the fact that net and type of injury exists between cold ischemia compared to warm ischemia [13].

Hepatocellular apoptosis decreased both with nitrite-supplemented Ringer's and UW solutions at 18 and 48 hours, respectively. There was less apoptosis during cold ischemia as compared to cold ischemia plus warm ischemia reperfusion. Nitrite treatment to only the liver graft or recipient mouse was more efficacious in reducing apoptosis compared to combination treatment of both graft and recipient. One possibility explaining this observation is that the observed “protective” concentrations of nitrite may have been exceeded leading to loss of protection per the U-shaped dose-response relationship. Further studies are required to determine optimal nitrite dosing and timing strategies to prevent liver injury during cold ischemia and posttransplantation. Liver grafts exposed to cold ischemia displayed only decreased caspase-3 and increased Bcl-2 with both Ringer's and UW's solutions supplemented with nitrite. The magnitude of change in both groups while divergent was enhanced with anion nitrite-supplemented UW solution.

Despite the study limitations that include a small sample size, no measurements of tissue concentrations of NO, and very short followup of the animals posttransplantation, in aggregate, our study demonstrated a hepatoprotective effect of nitrite as evidenced by reductions in liver enzymes, reduced pathology score, and reduced hepatocellular apoptosis with either LR or UW preservation solutions. The preservation solution, UW, afforded extended hepatoprotection compared to LR and nitrite. Liver grafts harvested from transplanted animals were observed to have reduced injury when only the graft or animal received nitrite supplementation compared to both graft and animal being supplemented with anion nitrite.
